# Deconstruction of Alkali Lignin and Lignocellulosic Substrates by *Aspergillus ochraceus* DY1 Isolated from Rotten Wood

**DOI:** 10.3390/jof10120810

**Published:** 2024-11-22

**Authors:** Namdol Nilza, Ram Prasad, Ajit Varma, Menaka Devi Salam

**Affiliations:** 1 Amity Institute of Microbial Technology, Amity University Uttar Pradesh, Noida 201313, India; 2 Department of Botany, Mahatma Gandhi Central University, Motihari 845401, India

**Keywords:** lignin degradation, *Aspergillus ochraceus*, rotten wood, alkali lignin, lignin valorization

## Abstract

The present study reports the ability of a fungal isolate *Aspergillus ochraceus* DY1, obtained from rotten wood, to degrade alkali lignin (AL) and lignocelluloses in an efficient manner. The efficiency of degradation was monitored by measuring the percentage of decolorization and utilizing GC-MS for identifying degradation products at different time intervals (10, 20, 30, and 40 days). The optimal degradation of alkali lignin (AL) was achieved at 0.01% concentration, 25 °C, and pH 7, resulting in 63.64% degradation after 40 days of incubation. A GC-MS analysis revealed significant degradation products, including n-hexadecanoic acid, octadecane, butylated hydroxytoluene, 2,6,11-trimethyl-dodecane, dibutyl phthalate, oleic acid, 3,5-dimethoxy-phenol acetate, and 2-(phenylmethylene)- cyclohexanone. Structural changes in AL were confirmed through HSQC 2D NMR and size-exclusion chromatography, indicating depolymerization and reduced molecular weight. Furthermore, *A. ochraceus* DY1 demonstrated substantial biomass loss in corn stover (62.5%) and sugarcane bagasse (50%) after 7 days of solid-state fermentation. Surface morphological depletion was observed in the bio-treated corn stover through SEM and confocal microscopy, which was not seen in the untreated one. These findings underscore the potential of *A. ochraceus* DY1 for efficient lignin degradation, with promising applications in biofuel production, waste management in the paper and pulp industry, and the synthesis of value-added bioproducts.

## 1. Introduction

Lignin makes up about 30% of the planet’s organic carbon and is the largest naturally occurring aromatic polymer [1]. In addition to the existence of rich aromatic compounds, one of the primary reasons for lignin’s importance is its availability, either directly from plants or as a by-product of industrial conversion operations. The major examples of these processes are the pulp and paper industries, as well as the lignocellulose feedstock biorefineries generating liquid fuels, such as bioethanol [2,3].

Several fungi and bacteria naturally break down this resistant branching polymer, and most of its products have commercial potential. These microbial lignin degradation pathways offer a wide range of alternatives to the problematic use of hazardous chemicals in the biodegradation of lignin waste in many industries [4]. Pulp and paper mills produce large amounts of wastewater containing lignin and face challenges in complying with environmental regulations. A number of treatment methods have been developed which rely on physico-chemical processes, but they cause environmental burdens and are costly. Therefore, efficient biological treatment methods are in demand [5]. Lignin recovery from the paper and pulp industries’ wastewater, and their conversion to valuable low molecular weight aromatic chemicals by exploiting potential microorganisms, has been under substantial focus during recent years. The recovery process is challenging as it tends to become modified during the pulping process. However, advanced technologies are being studied to obtain lignin from the wastewater of paper and pulp industries [6]. Therefore, in spite of its recalcitrant nature, lignin valorization is considered crucial for the full implementation of cost-effective lignocellulosic biorefineries [7].

Fungi, bacteria, and actinomycetes act together to degrade and alter lignin through biodegradation. Most of the wood-degrading fungi belong to *Basidiomycetes*, and significant studies have been conducted on their lignocellulolytic activity [8]. However, *Ascomycetes* that dwell in grasslands and shrublands are important determinants in carbon and nitrogen cycling as they are dominant in plant litter and soils. They may be present in associations with plants as endophytes, pathogens, or saprophytes in soil, playing a role in plant biomass decomposition [8]. Therefore, analyzing the lignin degradation potential of *Ascomycetes* and studying the molecular mechanism of lignin breakdown is necessary. Members of *Ascomycota* cause the soft rot of wood decay. Important examples are *Daldinia concentrica* [9] and *Podospora anserina* [10]. The genome analysis reports of some representatives of *Ascomycota* have shown the presence of many enzymes involved in lignin depolymerization. Moreover, genome and secretome analyses of the genera *Embellisia*, *Coniochaeta*, *Chaetomium*, *Phoma*, and *Aspergillus* sp. have shown the presence of genes encoding potential lignin degradation enzymes as well as the proteins and molecules involved in the decomposition of plants [8].

Extracellular lignin is broken down and modified by four different types of ligninolytic oxidative enzymes: laccase (Lac), manganese peroxidase (MnP), lignin peroxidase (LiP), and universal peroxidase [11]. The chemical compounds produced after the degradation of lignin are phenylacetic acid, 4-hydroxy-benzoic acid, vanillic acid [12], dibutyl phthalate [13], 3,5-dimethoxy-phenol derivatives, 2, 4-Di-tert-butylphenol, β-hydroxypropiovanillone [14], β-ketoadipate, p-cresol, 4-hydroxybenzoate, glucose, ethanol [15], β-ketoadipate enol-lactone, 3-oxohexanedioic acid, etc. Many of these degradation products are industrially important, and some of them have antioxidant properties. Hexadecenoic acid can be used in the production of surfactants as a natural additive in organic products, napalm, etc. Higher alkanes, like octadecane and nonane, are produced from lignin as by-products upon degradation and are some of the most important components of fuel oil and lubricating oil [16]. Dibutyl phthalate is a frequent lignin breakdown intermediate. The fungal peroxidase degradation of lignosulfonate produced the phthalate derivative bis(2-ethylhexyl) phthalate [17]. This shows that lignocellulosic waste can be converted into value-added products in a sustainable way with the help of lignin-degrading microbes.

The present study reports the assessment of *A. ochraceus* DY1 from rotten wood samples for lignin and lignocellulosic degradation. Its ability to depolymerize Alkali lignin (AL) has been analyzed in order to evaluate its potential biotechnological applications. In recent years, ligninolytic enzymes have gained applications mainly in lignin valorization to biofuel and high-value products, in pharmaceutical and cosmetics industries, and in the biodegradation of environmental pollutants containing phenolic compounds [18]. Since fungal ligninolytic enzymes have the capability of degrading different lignocellulosic substrates and producing high product yield in lignin valorization, the exploration of robust fungal lignin degraders is of interest for the study of their potential applications.

## 2. Materials and Methods

### 2.1. Isolation and Screening of Fungi

Soil samples were collected from the surroundings of the Haripur paper mill (geolocations: 30°91′29.431″ N and 76°84′77.035″ E) in Baddi, Himachal Pradesh, India, agricultural land (geolocations: 28°50′68.60″ N and 77°39′42.85″ E), and the biodiversity parks (geolocations: 28°53′55.17″ N and 77°39′10.29″ E) of Noida, Uttar Pradesh, India. Rotten wood samples were also collected from the biodiversity park and a wood warehouse in Noida, Uttar Pradesh, India. All samples were collected in sterilized bags and processed immediately after collection. Ten grammes of the soil sample were diluted in 90 mL of sterile 0.8% (*w*/*v*) saline water in a 250 mL conical flask and kept under a shaking condition at 150 rpm to obtain a homogenized soil suspension. The serial dilution of the soil suspension was performed aseptically up to 10^−5^ dilution. Following this, 0.1 mL from each dilution was spread plated onto a potato dextrose agar (PDA) medium containing 50 mgL^−1^ of chloramphenicol for the isolation of fungi. The inoculated plates were incubated at 27 °C for 5 to 7 days. For the isolation of fungi from rotten wood samples, decaying wood strips were surface sterilized and cut into pieces of 5 mm × 5 mm. The wood pieces were then plated on PDA media containing 50 mgL^−1^ of chloramphenicol and incubated at 27 °C for 7 days. Morphologically distinct isolates were selected and inoculated on fresh solid media and further screened for their lignin degradation potential on PDA containing different lignin representatives for screening, such as (i) 4 mM guaiacol, to detect laccase activity, where the formation of a brown colour zone around the colonies indicated positive ligninolytic activity; (ii) 1.5 mM aniline blue dye, to detect lignin peroxidase and manganese peroxidase activities through the decolourization of the dye; and (iii) 4 mM tannic acid, to detect tannase activity with the formation of clear zone. The plates were then incubated at 28 °C for 5 days and observed for any degradative activity. In addition, the degradation potential of lignocellulose was checked by screening the isolates on PDA plates supplemented with 0.5% carboxymethylcellulose (CMC) and 0.5% birchwood xylan for cellulase and hemicellulase activities, respectively.

### 2.2. Study of Alkali Lignin (AL) Removal Rate

To determine the removal rate of AL by the positive fungal isolate, it was inoculated in a minimal medium broth containing glucose 10 gL^−1^, KH_2_PO_4_ 0.5 gL^−1^, CaCl_3_ 0.075 gL^−1^, FeCl_3_ 0.01 gL^−1^, MgSO_4_ 0.15 gL^−1^, MnSO_4_ gL^−1^, and yeast extract 0.2 gL^−1^ with 0.02% (*w*/*v*) AL, and incubated at 27 °C for 7 days. The AL used in the experiment was from Tokyo Chemical Industry (India) Pvt. Ltd., Chennai, India (Product No. L0082) and is chemically processed and water-soluble. The content of the AL was measured at 280 nm and determined using the standard curve of AL. The removal rate of AL was determined using the formula, removal rate (%) = (C_1_ − C_2_)/C_1_ × 100, where C_1_ is the concentration of AL at t_0_ (i.e., before inoculation) and C_2_ is the concentration of AL after 7 days of fungal culture inoculation [19].

### 2.3. Identification of Most Potent Isolate

The isolate having the maximum AL removal was identified based on an internal transcribed spacer (ITS)-5.8S rRNA gene sequencing analysis. For molecular identification, genomic DNA was isolated using the AllPrep fungal DNA isolation kit (Qiagen, Hilden, Germany) and the purity and concentration were analyzed using a NanoDrop 2000 spectrophotometer. The ~700 bp ITS rDNA fragment was amplified using high-fidelity PCR polymerase, TaKaRa ExTaq (DSS TAKARA Bio India Pvt. Ltd., New Delhi, India). The primers used were: ITS5 Forward 5′ TCCGTAGGTGAACCTGCGG 3′ and ITS4 Reverse 5′ TCCTCCGCTTATTGATATGC 3′. The PCR cycling conditions were initial denaturation at 94 °C for 3 min, 30 cycles with denaturation at 94 °C for 1 min, annealing at 50 °C for 1 min, and extension at 72 °C for 2 min. The final extension was given at 72 °C for 7 min. The sequence data were aligned with available databases through NCBI BLAST, and the maximum homology of the sequence was checked.

### 2.4. Phylogenetic Analysis

The phylogenetic tree was constructed for the isolated strain using the MEGA 11.0 software [20] based on the ITS rDNA sequences and other types of strains of *A*. *ochraceus*. The neighbour-joining method was used with bootstrap values based on 1000 replications, where the scale was 0.02.

### 2.5. Effect of Different Parameters on Alkali Lignin Degradation

The effect of the AL concentration, pH, temperature, and incubation period on AL degradation was checked by varying the AL concentration (0.01%, 0.05%, 0.1%, and 0.5%), pH (3, 5, 7, and 9), temperature (22 °C, 25 °C, 28 °C, and 30 °C), and incubation period (10, 20, 30, and 40 days). The extent of the degradation was, thereafter, analyzed via FTIR, and the AL removal rate was calculated for each of the different conditions.

FTIR was performed to predict the unknown compounds present in the DY1-treated AL medium. The DY1-treated medium was centrifuged at 10,000 rpm for 5 min, after which the supernatant was collected in a fresh tube for FTIR testing in the Perkin Elmer FTIR/ATR instrument.

### 2.6. Analysis of Degradation Products

A GC-MS spectrometry was performed to identify the compounds present in the medium under optimized conditions of 10, 20, 30, and 40 days of incubation. For this study, the DY1-treated medium was centrifuged at 10,000 rpm for 15 min in falcon tubes, and the supernatant was mixed well with a 1:1 ratio of ethyl acetate for 30 min in a shaker. The contents were poured into a separating funnel and left for 15–20 min until the organic and inorganic layers separated. The lower inorganic layer was discarded, and the above organic layer was transferred into a flat dish for evaporation in a hot air oven until all the liquid evaporated. The contents were scraped out, mixed with methanol, and used for the identification of the compounds through GC-MS spectrometry. Agilent HP-5 ms Low Bleed GC/MS (30 m long, 0.25 mm inner diameter, 0.2 µm film thickness, particular number: 19091S-433) was used as a capillary column. Helium was used as the carrier gas at a flow rate of 1.3 mL/min. The column temperature was initially 50 °C for 1 min, then it was raised to 325 °C (10 °C/min, with a holding time of 5 min). The transfer line, quadrupole, and the ion source temperatures were 325 °C, 150 °C, and 250 °C, respectively. The solvent delay time was 4.50 min; the injector and detector temperatures were both 275 °C. One microliter of the methanol-soluble portion was directly injected, and the solvent delay time was 4.50 min. Mass spectra were recorded in the range of 150–510 *m*/*z*. The degraded products of lignin were identified by comparing mass spectra to the National Institute of Standards and Technology 11 (NIST17) library, which is available in the used instruments, i.e., GCMS-QP2010 Ultra (Shimadzu Corporation, Kyoto, Japan).

A 2D NMR was performed to predict the unknown compounds present in the DY1-treated AL medium. The fungal isolate, DY1, was incubated in a minimal medium broth containing glucose 10 gL^−1^, KH_2_PO_4_ 0.5 gL^−1^, CaCl_3_ 0.075gL^−1^, FeCl_3_ 0.01 gL^−1^, MgSO_4_ 0.15 gL^−1^, MnSO_4_ gL^−1^, and yeast extract 0.2 gL^−1^ supplemented with 0.01% (*w*/*v*) AL for 7 days at 25 °C and 160 rpm. The culture was filtered and then lyophilized; the content was collected in a fresh tube and dissolved with deuterated methanol for the 2D NMR testing. Two-dimensional NMR spectra were recorded at 25 °C in 5 mm tubes on a Bruker Avance II 300, Bruker, Billerica, MA, USA. Two-dimensional NMR spectra were recorded in HSQC (heteronuclear single-quantum correlation) spectroscopy.

Size exclusion chromatography (SEC) to determine the molecular weight distribution of the lignin before and after fungal treatment was performed on an Agilent 1260 Infinity GPC/SEC System (Agilent, Santa Clara, CA, USA) coupled to a UV detector and a RI detector. The control sample and the treated samples were dissolved in the solvent—tetrahydrofuran (THF) and filtered over a 0.45 μm syringe filter before injection. Five μL of the samples were injected into the SEC system with THF as eluent (0.5 mL min^−1^ at 40 °C). A standard calibration was performed with a set of narrow polydispersity pullulan standards for the molecular weight, ranging from 342 × 10^5^ to 3.44 × 10^5^ g mol^−1^, and polystyrene standards for the molecular weight, ranging from 162 × 10^6^ to 5 × 10^6^ g mol^−1^ (Agilent Technologies, Santa Clara, CA, USA).

### 2.7. Analysis of Bio-Treatment of Corn Stover and Sugarcane Bagasse

- Determination of biomass loss

Fungal treatment procedure

Corn stover and sugarcane bagasse were collected locally. They were crushed, washed, and dried in an oven. They were then ground, with the help of a laboratory grinding mill, into small particles of mesh, sized 1 to 2 mm, and stored in air-tight containers. The fungal treatment was carried out by inoculating the lignocellulosic substrate (corn stover or sugarcane bagasse), mixed with inorganic salt solution, having the compositions of 2 g L^−1^ (NH_4_)_2_SO_4_, 2 g L^−1^ KH_2_PO_4_, 0.3 g L^−1^ MgSO_4_, 0.3 g L^−1^ CaCl_2_, 0.5 g L^−1^ NaCl, 0.005 g L^−1^ FeSO_4_, 0.016 g L^−1^ MnSO_4_, and 0.017 g L^−1^ ZnCl_2_, in the ratio of 1:3 (*w*/*v*). A total of 3 mL of the spore suspension was inoculated into the medium under aseptic conditions and the mixture was cultivated for 21 days in static condition at 27 °C.

To check the biomass loss, the bio-treated corn stover or sugarcane bagasse was first regenerated via boiling at 100 °C for 15 min and the water was drained with the help of a filter paper. The sample was washed repeatedly with autoclaved distilled water until the water was visibly clear. Then, the corn stover was drained to get rid of excess water with the help of a muslin cloth, after which it was allowed to dry completely in an oven at 60 °C for 3 h until all the water weight was removed. After the biomass was completely dried, it was weighed to calculate the biomass loss according to the formula biomass loss = initial weight − final weight [21].

In order to check the correlation (if any) between biomass loss and the spore count, observations were conducted at regular intervals using the haemocytometer. A very tiny amount of bio-treated corn stover or sugarcane bagasse was taken in an Eppendorf tube, and 1 mL of distilled water was added to the tube. It was shaken well to transfer as many fungal spores into the liquid as possible. A total of 100 μL of the suspended liquid was placed on the haemocytometer and a cover slip was placed on top. The haemocytometer was then observed under a microscope. This observation was made in two-day intervals for a period of 23 days [22].

- A microscopic analysis of bio-treated corn stover and sugarcane bagasse

The regenerated biomass samples, i.e., the untreated and the DY1-treated, which were boiled and then washed several times with autoclaved distilled water, were finally dried in a hot air oven at 50 °C for 2 h. A few strands of the biomass samples were mounted on the coverslip and coated with poly L-lysine [23]. The sample was then dehydrated with methanol and kept at room temperature for drying. The dried samples were mounted on aluminium stubs, sputter coated with a thin layer (6 nm) of gold. The samples were analyzed through a scanning electron microscope (ZEISS EVO, Jena, Germany) with a beam voltage of 20 kV. The surface morphologies of the untreated and the bio-treated corn stover and sugarcane bagasse were compared.

The samples were also analyzed under a confocal scanning laser microscopy (CSLM). Very thin-sectioned samples were distributed on the glass microscope slide and covered with glass coverslips. Images were captured using Nikon’s Confocal microscope A1 under 60× magnification at Amity Institute of Microbial Technology, Amity University, Noida, India, and analyzed using NIS Elements software (NIS-Elements AR 6.02.01) (Nikon, Tokyo, Japan).

### 2.8. Ligninolytic Activity

A total of 5 g of each substrate (corn stover and sugarcane bagasse) was taken in 250 mL conical flasks. Each substrate-containing flask was moistened with 5 mL of distilled water and sterilized by autoclaving at 121 °C for 20 min. A conidial suspension was prepared by mixing two 5 mm mycelial discs from a 7-day-old strain in 10 mL of sterile water achieving 2 × 10^−6^ spores/mL. The substrates were then inoculated in 3 mL of the spore suspension under aseptic conditions and incubated for 14 days in static conditions at 27 °C. For the extraction of the crude enzyme, the experimental flasks were filled with 50 mL of autoclaved distilled water and placed in a shaker incubator at 120 rpm for an hour. Using Whatman No. 1 filter paper, the biomass was individually filtered, and the filtrate was thereafter centrifuged at 5000 rpm for 20 min. The supernatant was used as a crude enzyme for testing ligninolytic enzyme activity. One ml of the supernatant was mixed with 1 ml of 2 mM guaiacol, along with 3 mL of the 10 mM sodium acetate buffer (pH 5), in a test tube. A blank was also prepared, which contained 1 ml of distilled water instead of the culture supernatant. The mixtures were incubated for 15 min at 30 °C and the absorbance read 450 nm using a UV-vis spectrophotometer. The laccase activity was determined by observing the absorbance at 450 nm (ε = 0.6740 μM/cm). One unit of laccase activity was expressed as the amount of enzyme required to oxidize 1 µmol of guaiacol per minute [24].

## 3. Results

### 3.1. Isolation and Screening of Microorganisms

Out of 22 morphologically distinct fungal isolates, eight isolates showed lignin degradation and/or lignocellulose degradation potential during preliminary screening. The fungal isolate, DY1, showed the best degrading activities on the PDA containing different lignin representatives for screening, i.e., guaiacol, aniline blue dye, and tannic acid (Table 1). The zone of decolourization and oxidation activity was observed around the culture within 48 h of incubation for the isolate DY1. Moreover, it showed positive results in the screening of cellulase and xylanase activities.

### 3.2. Study of Alkali Lignin Removal Rate

The lignin degradation ability was determined by the alkali lignin removal rate (%). The DY1 isolate, which was selected based on the preliminary screening results, was grown in a medium containing AL as the sole carbon source and the remaining lignin content after incubation was determined using a UV-spectrophotometer at 280 nm. The percentage of AL removal rate was determined to be 76.65% after 7 days of incubation.

Removal rate (%) = (C_1_ − C_2_)/C_1_ × 100

= (1.89 − 0.441)/1.89 × 100

= 76.65%

### 3.3. Identification of the Most Potent Isolate

The molecular identification of the fungal isolate DY1 was carried out based on ITS region sequencing. A total length of 593 bp of the ITS region was sequenced, and it was found that the strain had 99–100% homology, with various strains of *A. ochraceus*. Therefore, the fungal isolate in the present study was designated as *A. ochraceus* strain DY1. The Genbank accession number for the ITS region of strain *A*. *ochraceus* DY1 is OP804322.

### 3.4. Phylogenetic Analysis

**Figure 1:**
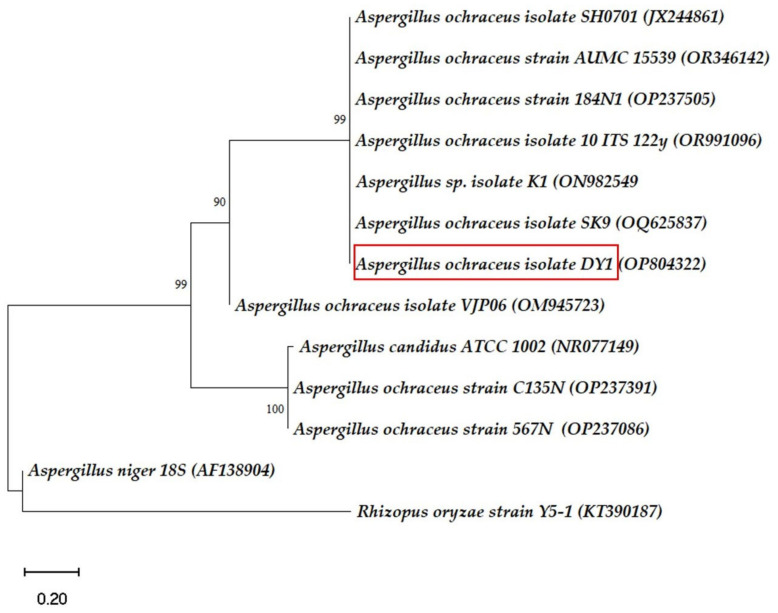
Phylogenetic tree of isolates based on ITS region sequencing. The isolate in the present study is highlighted with a red colour box. The NCBI accession numbers of the respective sequences are given in parentheses after the microorganism names.

To determine the relationship between *A*. *ochraceus* DY1 (Taxonomy ID: 40380) and 11 other related strains of *Aspergillus* sp. based on sequence analyses, a phylogenetic tree was obtained using MEGA11. The *Aspergillus niger* strain 18S and *Rhizopus oryzae* strain Y5-1 were included as members other than *A. ochraceus*, and as non-ascomycete strains, respectively. It was observed that the nearest neighbours with the same evolutionary branch were six different strains of *A. ochraceus* with bootstrap value 99 (Figure 1). These strains also showed 99% to 100% similarities with *A. ochraceus* DY1 in the BLAST analysis. A few strains of *A. ochraceus* chosen during the phylogenetic tree construction appeared under separate nodes from the ones under study and, therefore, have different evolutionary branches. They were: (i) *A. ochraceus* isolate VJP06, as a separate node with the bootstrap value 90, and two other strains, *A. ochraceus* strain C135N and *A. ochraceus* strain 567N, which were related to *A. candidus* ATCC 1002, as a separate node with bootstrap value 100; all of them arise from a common ancestor. *Aspergillus niger* and the *Rhizopus oryzae* strain Y5-1 appeared under separate evolutionary branches.

### 3.5. Effect of Different Parameters on AL Degradation

#### 3.5.1. Effect of Lignin Concentration on AL Degradation

**Figure 2:**
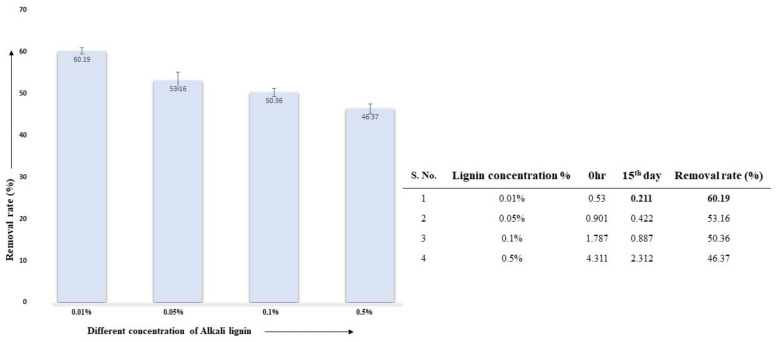
Effect of different lignin concentrations on their degradation via *A. ochraceus* DY1. The results are the mean of three distinct replicates, with standard deviation represented by error bars.

Lignin degradation, using the selected fungal isolate *A. ochraceus* DY1 at different concentrations of AL, was studied. The decolourization (degradation) was not observed in the control, i.e., the uninoculated medium supplemented with AL. The test sample with 0.01% AL had the highest lignin degradation, as calculated by the percentage removal rate at 60.19%, followed by 0.05%, 0.1%, and 0.5% lignin concentrations, as shown in Figure 2.

**Figure 3:**
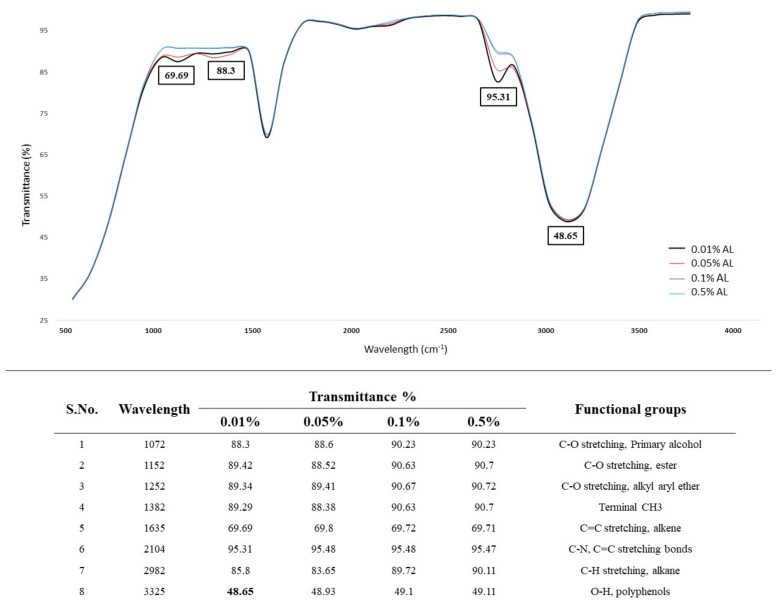
FTIR spectra of the effect of different AL concentrations on their degradation via *A. ochraceus* DY1. The AL concentrations used were 0.01%, 0.05%, 0.1%, and 0.5%, represented by different colour lines in the spectra. The transmittance values where major peaks were detected are indicated inside the boxes. The wavelengths where peaks were detected, and the corresponding functional groups, are shown below the spectra.

The results of the FTIR analysis of AL treated with *A. ochraceus DY1* are shown in Figure 3. More new peaks appeared in the treated sample compared to the untreated one, indicating changes in the functional groups of the products formed during the fungal treatment of AL. A characteristic broad peak of an aliphatic alcohol with an O–H stretch at 3325 cm^−1^ and an C–O stretch at 1072 cm^−1^ was observed in the spectrum. The C–O stretching, which is a sharp peak, also supports obtaining a phenol structure. The peaks at 2982 cm^−1^ and 1635 cm^−1^ indicate the presence of the C–H stretching of the alkane group and the C=C stretching of alkene (monosubstituted), respectively. The peak at 1382 cm^−1^ shows O–H bending, which confirms the phenol structure. The peaks at 1152 and 1252 cm^−1^ show C–O stretching and the peak at 2104 cm^−1^ shows C–N and C=C stretching, as shown in Figure 3.

#### 3.5.2. Effect of Temperature on AL Degradation

**Figure 4:**
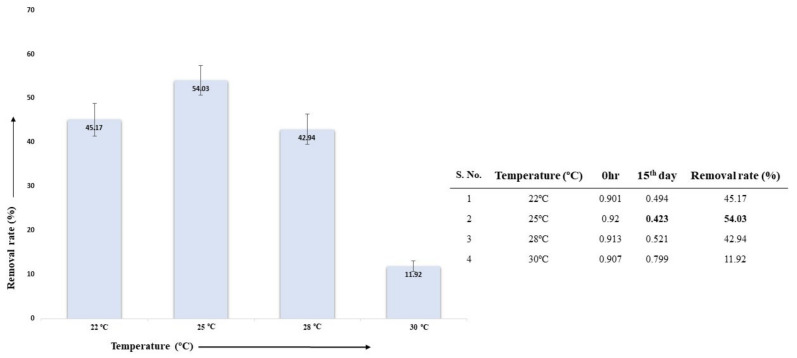
Effect of temperature on lignin degradation via *A. ochraceus* DY1. The results are the mean of three distinct replicates, with standard deviation represented by error bars.

Lignin degradation via the selected fungal isolate *A. ochraceus* DY1 at different temperatures with 0.05% (*w*/*v*) AL was studied. The sample incubated at 25 °C had the highest percentage of lignin degradation at 54.03%. The difference in the percentage removal rate at 25 °C from the previous experiment that showed 60.19% might be due to the percentage of lignin concentrations used. At 22 °C, 28 °C, and 30 °C, the degradation percentages were 45.17%, 42.94%, and 11.92%, respectively, as shown in Figure 4.

**Figure 5:**
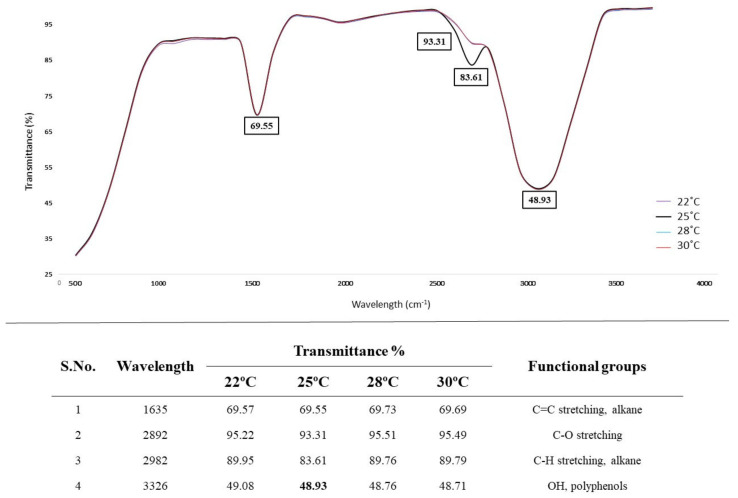
FTIR spectra of the effect of temperature on AL degradation via *A. ochraceus* DY1, shown as different colours for the temperatures 22 °C, 25 °C, 28 °C, and 30 °C. The values inside the boxes indicate transmittance values of the peaks. The wavelengths where peaks were obtained, and their corresponding functional groups are shown below the spectra.

The result of the FTIR analysis of AL treated with *A. ochraceus* DY1 at different temperatures is as shown in Figure 5. There was no significant difference between the different temperatures, but the peaks obtained at 1892 cm^−1^ and 1982 cm^−1^, which are of C=O stretching C–H stretching and alkane, respectively, were sharper and more prominent at 25 °C.

#### 3.5.3. Effect of pH on AL Degradation

**Figure 6:**
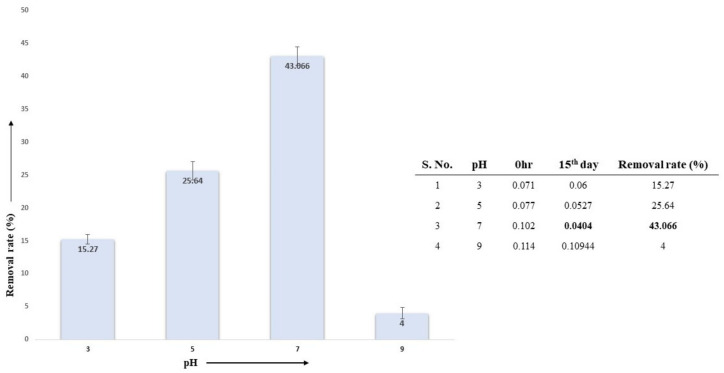
Effect of different pH on lignin degradation via *A. ochraceus* DY1. The results are the mean of triplicates, with standard deviation represented by error bars.

Lignin degradation via the selected fungal isolate *A. ochraceus* DY1 at different pH levels was studied. The sample with pH7 had the highest percentage of lignin degradation at 43.07%, whereas pH3, pH5, and pH9 showed 15.27%, 25.64%, and 4%, respectively, as shown in Figure 6.

**Figure 7:**
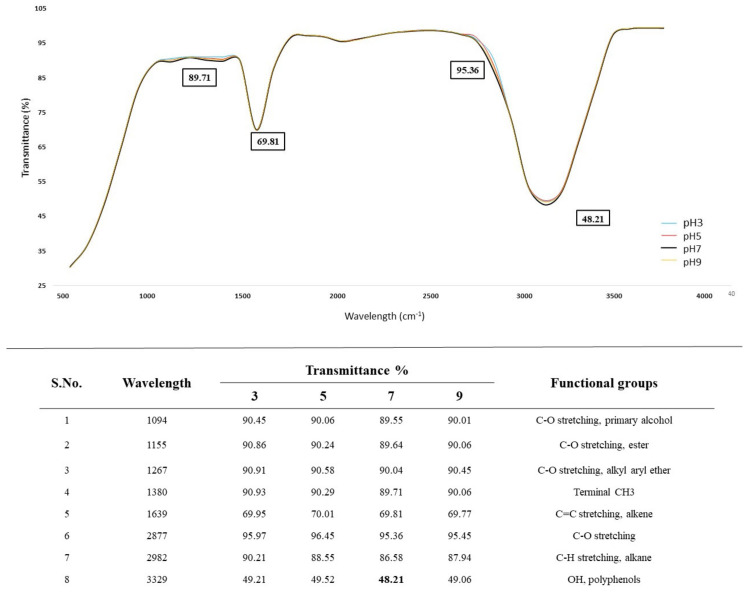
FTIR spectra of the effect of pH on AL degradation via *Aspergillus ochraceus* DY1, as shown by different colour spectra for the pH values 3, 5, 7, and 9. The values inside the boxes indicate the transmittance values of the peaks. The absorption peaks and their corresponding functional groups are shown below the spectra.

The results of the FTIR analysis of AL treated with *A. ochraceus* DY1 at different pH are shown in Figure 7. There was no significant difference between the peaks obtained at different pH, although the decolourization of AL was best obtained at pH7.

#### 3.5.4. Effect of Incubation Period on AL Degradation

**Figure 8:**
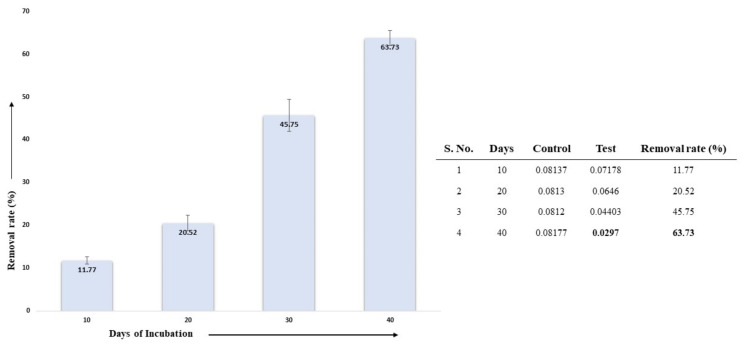
Effect of different days of incubation on lignin degradation by *Aspergillus ochraceus* DY1. The results are the mean of triplicates with error bars representing standard deviation.

The effect of different days of incubation on the lignin degradation in the presence of the selected fungal isolate *Aspergillus ochraceus* DY1 and 0.01% (*w*/*v*) AL was studied. The sample incubated for 40 days had the highest lignin degradation with 63.73%, whereas the sample incubated for 10 days showed an 11.77% lignin concentration; 20 days and 30 days of incubation showed 20.52% and 45.75%, respectively. This indicates that with gradual increases in the incubation period, the degradation of lignin also increases, as shown in Figure 8.

**Figure 9:**
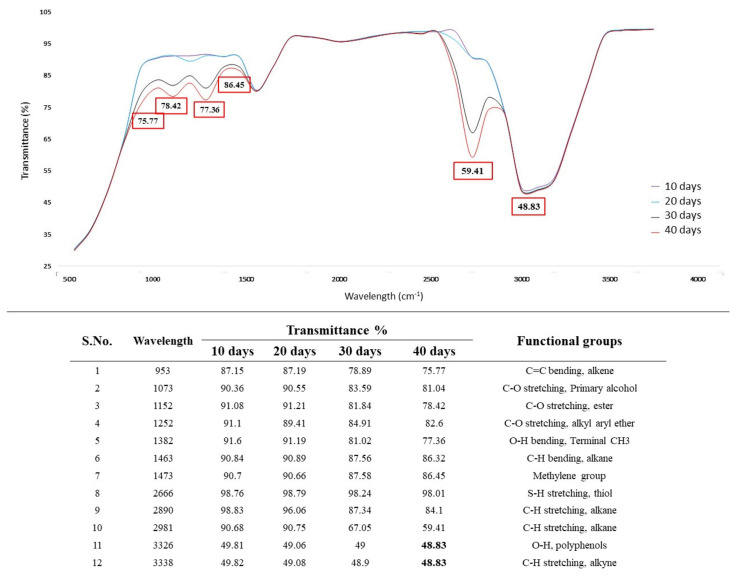
FTIR spectra of the effect of incubation time on AL degradation via *Aspergillus ochraceus* DY1 shown as different colour spectra for 10, 20, 30, and 40 days of incubation. The values inside the boxes indicate the transmittance values of the peaks. The absorption peaks and their corresponding functional groups are shown below the spectra.

The results of the FTIR analysis of AL treated with *Aspergillus ochraceus* DY1 at different incubation periods are shown in Figure 9. The incubation period of 40 days gave peaks which were sharp and significant compared to the 10- and 20-day incubation periods. The prominent peaks were at 2981 cm^−1^ (C–H stretching, alkane), 1382 cm^−1^ (O–H bending, terminal -CH_3_), 1152 cm^−1^ (C–O stretching, ester), and 953 cm^−1^ (C=C bending, alkene). These peaks were not visible during the 10- and 20-day incubation periods.

### 3.6. GC MS Analysis of AL Degradation

The lignin degradation potential of *Aspegillus ochraceus* DY1 was further checked by treating the alkali lignin with the strain and analyzing the degradation products on different days using GC MS. Technical lignin may contain different functional groups, such as phosphate, sulphonate, butyl groups, etc., depending on the process and chemical reactions utilized.

The degradation products of AL by *Aspergillus ochraceus* DY1 were analyzed by GC-MS. The results of untreated and treated AL at different days of incubation are shown in Table 2. The possible degradation products are n-hexadecanoic acid, oleic acid, octadecane, butylated hydroxytoluene, 2,6,11-trimethyl-dodecane, dibutyl phthalate, 3,5-dimethoxy-phenol acetate, and 2-(phenylmethylene)-cyclohexanone, which were produced during the incubation kept up to 40 days. The larger compounds linked to the technical lignin or processed lignin structure, which gradually disappeared during the incubation period, were 13-(2-Methoxyphenyl) tricyclo[8.2.2.24,7] hexadeca-1, phenol, 2,4-bis(1,1-dimethylethyl)- phosphite, 4,4’-butylidenebis[2-(1,1-dimethylethyl)-5-methyl- phenol, and 7,9-Di-tert-butyl-1-oxaspiro (4,5) deca-6,9-diene-2,8-dione.

**Figure 10:**
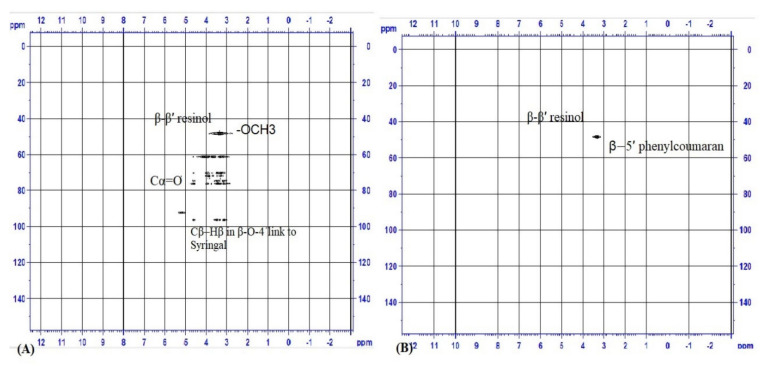
2D HSQC NMR characterization of (**A**) untreated AL and (**B**) DY1-treated AL.

The HSQC 2D NMR spectroscopy analysis was carried out to demonstrate the structural characteristics of compounds generated after the treatment of AL with *Aspergillus ochraceus* DY1, as shown in Figure 10. The structural changes in the depolymerization process were clearly demonstrated in the comparison between the fresh AL and the DY1-treated AL. It can be clearly seen from this figure that the untreated AL had δ_C_/δ_H_ = 50.0–60.0/3.0–4.0, indicating the Cβ-Hβ in β-β’ resinol structures, the β-5’ in phenylcoumaran structures, and the C–H in -OCH3 structures. The δ_C_/δ_H_ = 60.0–80.0/3.0–4.0 indicates the Cγ-Hγ in β-β’ resinol structures and the δ_C_/δ_H_ = 60.0–80.0/4.0–5.0 indicates Cα=O in Hibbert ketone. The cross-peaks of Cβ–Hβ in β-O-4’substructures linked to syringyl units were indicated by the chemical shift of δ_C_/δ_H_ = 80.0–100.0/4.0–5.0. When compared with the spectra of the untreated AL, the spent lignin after fungal treatment showed only the δ_C_/δ_H_ 40.0–60.0/3.0–4.0, indicating the β-β resinol and β-5 phenyl coumaran structures of lignin. The difference in the ^13^C–^1^H correlation signals between fresh and spent AL, and also the disappearance of β-O-4’aryl ether linkages, show the process of depolymerization in the fungal-treated AL. 

SEC was performed to observe the degradation of AL by comparing the changes in the molecular weight (Mw) distribution of the polymer after it was treated with *Aspergillus ochraceus* DY1. In this study, the Mw of AL, before and after 7 days of incubation with strain DY1, was evaluated. The average Mw of the sample on day 0 was approximately 15,776.2 Da. The Mw of the treated sample decreased to 12,219.2 Da on the 7th day after being treated with *Aspergillus ochraceus* DY1. In addition, the polydispersity (Mw/Mn) of lignin fragments decreased from 2.2 to 1.67 on day 7. A decrease in the molecular weight distribution of treated AL over time indicated that AL was successfully degraded after 7 days of treatment with *Aspergillus ochraceus* DY1 (Table 3).

### 3.7. Analysis of Bio-Treatment of Corn Stover and Bagasse

**Figure 11:**
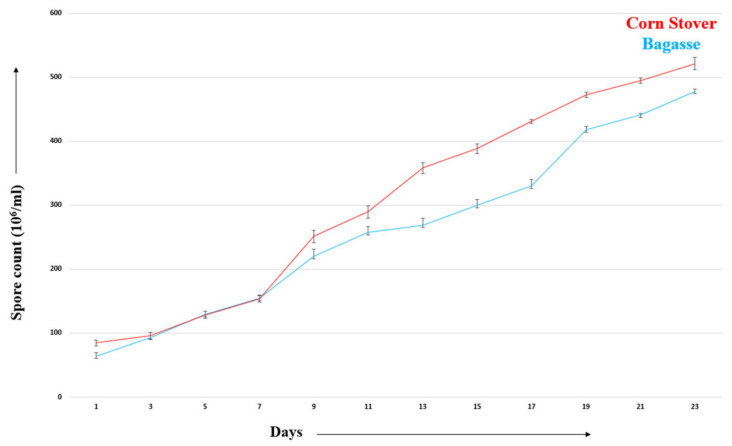
Spore count of *Aspergillus ochraceus* grown on lignocellulosic biomass.

*Aspergillus ochraceus* DY1 was analyzed for its potential applications in the bio-treatment of corn stover and sugarcane bagasse. In SSF using 4 g of corn stover, biomass loss of corn stover after 7 days of treatment was found to be 62.5%, indicating the consumption of corn stover during bio-treatment. Similarly, when sugarcane bagasse was used, the biomass loss was observed to be 50%. In correlation, a gradual increase in the spore count of *A. ochraceus* DY1 was observed during 23 days of incubation in corn stover and in sugarcane bagasse at 27 °C (Figure 11).

### 3.8. Microstructure Analysis of Bio-Treated Corn Stover

**Figure 12:**
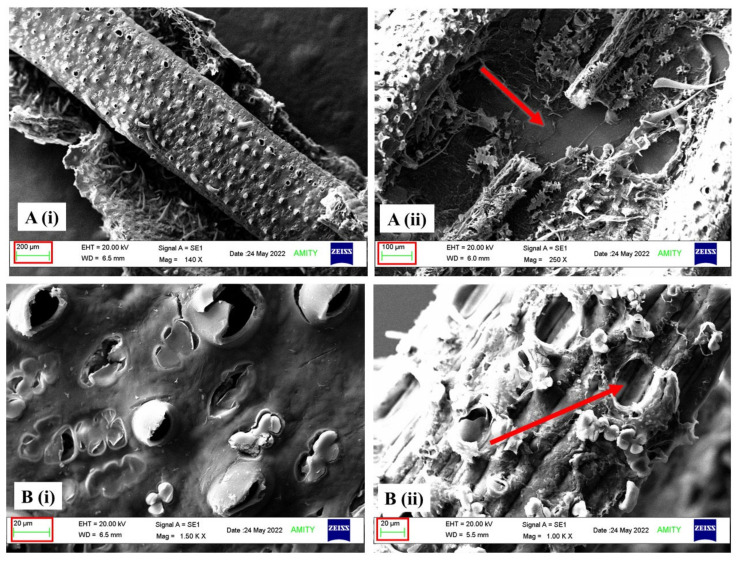
SEM of untreated and pre-treated corn stover. (**A**) (**i**) and (**B**) (**i**) untreated corn stover, (**A**) (**ii**) and (**B**) (**ii**) bio-treated corn stover. The red colour arrows indicate pits and disintegration of structural morphology in the bio-treated corn stover.

**Figure 13:**
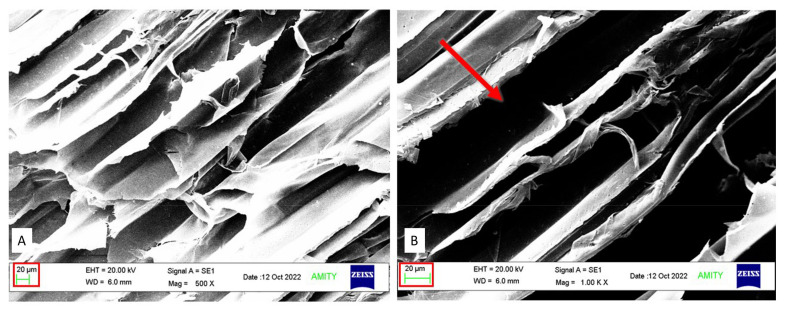
SEM of (**A**) untreated, and (**B**) bio-treated sugarcane bagasse. The red colour arrow indicates surface disintegration in the bio-treated sugarcane bagasse.

**Figure 14:**
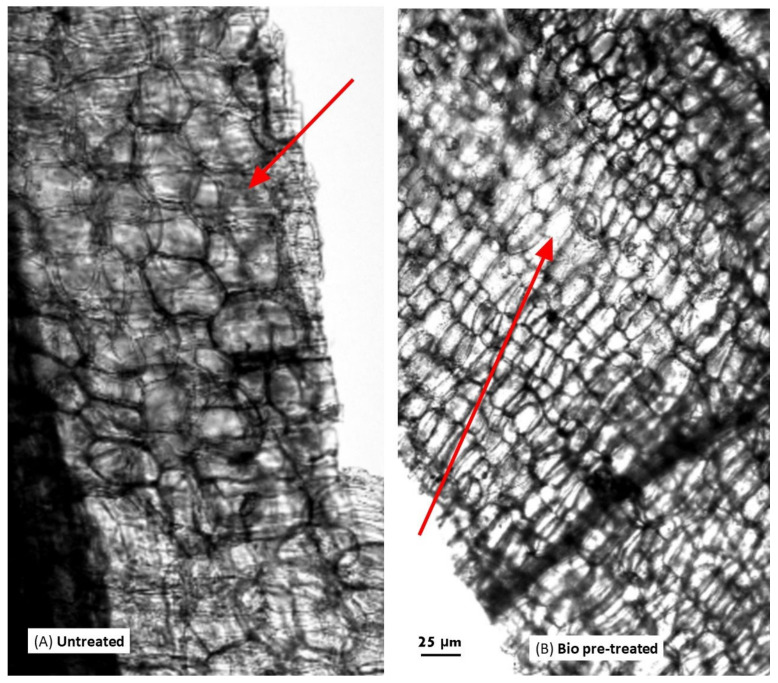
Surface morphologies of the (**A**) untreated (control), and (**B**) bio-treated corn stover observed by microscopy. Scale bar: 25 µm. The red colour arrows indicate smooth surface with thick layering, and rough surface with thin layering in the untreated and bio-treated cornstovers respectively.

Structural analyses of bio-treated corn stover and sugarcane bagasse were carried out by scanning electron microscopy (SEM), and it was found that there was a significant depletion of structural morphology (Figure 12 and Figure 13). The surface of untreated corn stover showed smooth and uniform structural integrity, whereas DY1-treated corn stover showed various pits and disintegration, demonstrating that the corn stover surface was partially destroyed. SEM results were also supported by confocal microscopy results. The representative image of the deconstruction of the lignocellulosic raw material DY1 is shown in Figure 14, where the bio-treated corn stover is seen to have a rough surface with thin layering compared to the untreated one, which showed a smooth surface with thick layering under confocal microscopy. This is because of the degradation or removal of layers by the fungal treatment. These morphological changes indicated that degradation occurred on the surfaces of both corn stover and sugarcane bagasse after bio-treatment due to the degradation of lignocellulose by the enzymes secreted by the fungus DY1 during growth in biomass.

### 3.9. Ligninolytic Activity

The extracellular laccase-like activity was detected through the significant colour change from brownish yellow to reddish brown in the assay tubes containing culture supernatant mixed with 2 mM guaiacol dye along with the 10 mM sodium acetate buffer. The laccase-like activity was calculated to be 0.187 UmL^−1^ and 0.168 UmL^−1^, respectively when corn stover and sugarcane bagasse were used as raw material substrates. The control sample in which boiled culture supernatant was used, in order to denature the enzyme, did not show any colour change. Also, a reaction mixture containing 5 mM L-cysteine as a laccase inhibitor was used, which showed no colour change, indicating that the enzyme may be a laccase.

## 4. Discussion

The study in this paper aimed to screen potential fungi collected from rotten wood samples and wood warehouses that could possibly degrade lignin. Primary screening for ligninolytic activity through the qualitative plate assay method using aniline blue dye, guaiacol, and tannic acid showed that the fungal isolate DY1 had the maximum capability to utilize the lignin model compounds within 48 h of incubation. Furthermore, it was found that the DY1 isolate could degrade CMC and birchwood xylan when screened on a PDA medium supplemented with its respective substrates. This indicates that the isolate has cellulase and xylanase activities, which are important for the degradation of lignocellulosic biomass. The fungal isolate DY1 was identified as *A. ochraceus* DY1 through morphological and 5.8S rDNA/ITS analyses. The phylogenetic tree generated using MEGA11, with a scale bar representing 0.01 base substitutions per site and bootstrap values indicating branch reliability, shows that *A. ochraceus* DY1 (OP804322.1) clusters are closely aligned with other *A. ochraceus* strains, confirming its identification as a member of this species. Lignin-degrading microorganisms have been reported from various sources such as soil samples and effluent discharge [25], commercial coir [26], pulp and paper mill wastewater [27], forest environment [28], decayed woods [29], etc. Based on previous reports, *Pseudomonas* sp., *Ochrobactrum* sp., and *Burkholderia* sp. have high ligninolytic enzyme activity among bacteria. Whereas in the case of fungi, white-rot fungi, such as *Phanerochoete chrysosporium*, *Phlebia radiata*, *Coriolus versicolor*, *Bjerkandera adusta*, and *Panus tigrinus*, etc., have been reported as high lignin-degrading fungi [30]. Wood-decaying fungi mostly belong to *Basidiomycetes*, including some members of *Ascomycetes*. According to [31], about 10,000 species of fungi causing white rot and having lignocellulose degrading activity are estimated to exist, but they have not been fully explored. In the present study, *A. ochraceus* DY1 has been found to exhibit laccase-like activity, a typical characteristic of the *Ascomycetes* group of soft-rot fungi, according to [32]. This result is supported by the presence of their corresponding genes through the whole genome analysis of *A. ochraceus* DY1 [33]. When grown in a minimal medium and having alkali lignin as the sole carbon source, it was able to decolourize it significantly with a removal rate of 76.65% in 7 days. In previous reports, *Lentinus edodes* (UEC-2019) and *Basidiomycetes* were reported to remove 73% of AL in 5 days [34]. *Trametes hirsuta* X-13, another *Basidiomycetes* which causes white rot, was able to decolorize AL up to 50% in 7 days [35]. *Phanerochaete chrysosporium*, the white-rot lignin-degrading fungus, was reported to degrade up to 90% of AL within 7 days by performing the spectrophotometric method of lignin assay [36]. Also, *Aspergillus flavus* and *Emericella nidulans* were reported to degrade AL by 14.4% to 21% in different media by measuring the decolourization percentage and residual AL using UV-visible spectroscopy [25]. Su et al., 2018, studied 63 strains for the degradation of AL and the highest degradation ability was reported to be 36.73% in 4 days by an isolate identified as *Myrothecium verrucaria*. The AL degradation was assayed as a percentage removal rate of AL using the UV spectroscopy method [37]. The FTIR analysis of the degraded sample showed peaks corresponding to C–H bending at 1463 cm^−1^ (alkane), C=C bending at 953 cm^−1^ (alkene), C–O stretching at 1252 cm^−1^ and at 1152 cm^−1^ (alkyl-aryl ether, ester), O–H bending at 1382 cm^−1^ (terminal -CH3) as new peaks apart from the peak at 3326 cm^−1^, which is of polyphenols. This indicated the deconstruction of AL by the strain when checked up to 40 days of incubation in the AL medium.

In the present study, many low molecular weight acidic and aromatic compounds were identified in the extract of the degraded sample. The compounds, such as hexadecenoic acid, dibutyl phthalate, butylated hydroxytoluene, 3,5-dimethoxy-phenol acetate, and 2-(phenylmethylene)-cyclohexanone, were detected after 20 days of incubation, indicating that they might be the compounds produced through AL degradation. The compounds, such as 13-(2Methoxyphenyl) tricyclo[8.2.2.24,7]hexadeca-1, Phenol, 2,4-bis(1,1-dimethylethyl)-, phosphite, Phenol,4,4’-butylidenebis[2-(1,1-dimethylethyl)-5-methyl, appeared in the initial period of incubation, i.e., up to 20 days, and then disappeared during the later stages, indicating that they belong to the structure of the technical lignin used in the experiment. The compound 7,9-Di-tert-butyl-1-oxaspiro (4,5) deca-6,9-diene-2,8-dione was detected throughout the experiment, which shows the recalcitrant part of the structure. The study by Chandra and Bhargava (2013) reported derivates, such as tetradecanoic acid, hexadecanoic acid, 1-phenathrene carboxylic acid, and Bis (2-ethylhexyl) phthalate, after the efficient degradation of AL via mixed bacterial cultures *Citrobacter freundii* (FJ581026) and *Citrobacter* sp. (FJ581023) at pH 8, temperature 35 °C, 600 ppm AL, and 6 days of incubation. The cultures were reported to produce manganese peroxidase [38]. Contrary to the result in the present study, Riyadi and his team reported the production of 3-methyl-butanoic acid, guaiacol derivatives, and 4,6-dimethyl-dodecane from 2.5 g/L Kraft lignin treated with *Streptomyces* sp. strain S6 at 30 °C for 7 days. The ligninolytic enzymes detected were lignin peroxidase, laccase, dye-decolorizing peroxidase, and aryl-alcohol oxidase activities [17]. This variation in the result may be due to the difference in the type of lignin substrates used in the experiments. A study carried out by Cui and his team showed 23 lignin degradation products as detected via GC-MS, which included n-hexadecanoic acid, oleic acid, octadecane, butylated hydroxytoluene and 2,6,11-trimethyl-dodecane [39]. The lignin degradation percentage was reported to be 50%. In another study, lignin monomer compounds like 2-methoxy phenol, 2,6-di-methoxy phenol and its alkylated derivatives were found in the degraded alkali lignin which showed 19–41.6% degradation in 21 days [25]. Xu et al., 2022, reported vanillic acid and 3,5-dimethoxy-4-hydroxycinnamaldehyde through a GC-MS analysis of alkali lignin degradation, which only showed 8 to 14% degradation [40]. The production of 15 elements (guaiacol, phenylacetic acid, benzene, benzoic acid, 4-hydroxyacetophenone, 4-allyl-2-methoxyphenol, (3-ethoxy-4-hydroxyphenyl) (hydroxy) acetic acid, vanillin) has been reported from AL, having 40% degradation in 7 days [41]. The difference in the lignin degradation profile may be due to the difference in the degradation rate. The present strain, *A. ochraceus* DY1, has an efficient degradation capability of 76.65% degradation in 7 days. Confirmation of the depolymerization process was obtained by the results of the SEC and 2D HSQC NMR analysis. In SEC, the polydispersity of lignin fragments decreased from 2.2 to 1.67 on day 7, and a decrease in the molecular weight distribution of treated AL over time indicated that AL was successfully degraded after 7 days of treatment with *A. ochraceus* DY1. In 2D HSQC NMR, the spent AL sample showed an absence of important linkages that are supposed to be present in the lignin structure. The 2D HSQC of untreated AL showed the signal characteristics of technical lignin (lignin obtained as byproducts from the pulping industry), in which phenylglycerol structures, such as guaiacylglycerol (G-gly) and syringylglycerol (S-gly), were detected at δC/δH = 73.2–75.2/4.3–4.5, 74.7–76.9/3.4–3.6, and 61.2–65.6/3.1–3.8. Similar results were observed in a study on the structural analysis of technical lignins obtained after the pulping process. The other signals detected in the 2D HSQC of untreated AL were Cβ-Hβ in β-β’ resinol structures, the Cβ–Hβ in β-O-4’substructures linked to syringyl units, Cγ-Hγ in β-β’ resinol structures, the β-5’ in phenylcoumaran structures, the C–H in -OCH3 structures, and the Cα=O in Hibbert ketone. When compared with the spectra of the untreated AL, the spent lignin after fungal treatment showed only the δC/δH 40.0–60.0/3.0–4.0, indicating the β-β resinol and β-5 phenyl coumaran structures of lignin. Depolymerization in the fungal-treated AL was confirmed by the disappearance of β-O-4’aryl ether linkages and by most of the other signals detected in the untreated AL. Similar results were obtained in previous studies involving SEC and 2D HSQC NMR studies, which contained comparisons between depolymerized and non-depolymerized lignin samples [21].

*A. ochraceus* DY1 was also observed to utilize lignocellulosic biomass, such as corn stover and sugarcane bagasse, efficiently, as shown by the lignocellulose biomass loss of 62.5% and 50%, respectively, within 7 days of incubation. There was a corresponding increase in the spore count of DY1, which correlates with the biomass utilization. Further microscopic analyses of the treated biomass, i.e., corn stover and the sugarcane bagasse, showed depletion in the structural integrity of the biomasses compared with the untreated control. A similar observation was reported in a study of the treatment of corn stover by *Ceriporiopsis subvermispora* and *Phanerochaete chrysosporium*, in which small but significant changes in the cross sections of the parenchyma cells and vascular bundles were observed under SEM after 2 weeks of incubation [42]. Su et al., 2018 also reported similar results while studying the bio-pretreatment of corn stover by *Myrothecium verrucaria* [37]. This shows the potential of the fungal strain in the present study for its application in the biotreatment of agricultural waste biomass for sustainable waste management.

## 5. Conclusions

The present study reports the deconstruction of AL by *A. ochraceus* DY1 and its ability to consume lignocellulosic substrates, corn stover, and sugarcane bagasse, with the production of laccase-like activity. The conditions for the maximum removal of AL in a minimal medium were found to be 0.01% AL, 25 °C, pH 7, and an incubation period of 40 days. Important compounds, such as n-hexadecanoic acid, oleic acid, octadecane, butylated hydroxytoluene, 2,6,11-trimethyl-dodecane, dibutyl phthalate, 3,5-dimethoxy-phenol acetate, and 2-(phenylmethylene)-cyclohexanone were detected when the AL containing medium was inoculated with the strain under optimized conditions. This shows the ability of *A. ochraceus* DY1 to utilize AL and transform it into valuable compounds. AL is a by-product of the pulp and paper industry, and the use of microorganisms to treat the wastewater and to convert the recovered lignin into value-added chemicals has been of interest in recent years. *A. ochraceus* DY1 can also be further used to study the large-scale production of ligninolytic enzymes using lignocellulosic waste. The ability of this strain to grow efficiently in a medium containing AL as the sole carbon source and in lignocellulosic materials shows its potential applications in the treatment of industrial and agricultural wastes after carrying out careful investigations on how it can be applied for achieving efficient outcomes.

## Figures and Tables

**Table 1:** Status of various fungal isolates on different indicators of lignin biodegradation.

Isolate	Sample Type	Source	Indicator			CMC Hydrolysis	Birchwood Xylan Hydrolysis
			Guaiacol	Aniline Blue Dye	Tannic Acid		
SL4	Soil	Agricultural field	0.2 mm	-	-	0.3 mm	0.5 mm
SL5	Soil	Agricultural field	0.3 mm	0.6 mm	-	1 mm	-
PS12	Soil	Paper mill	2.3 mm	0.9 mm	0.8 mm	0.6 mm	0.8 mm
PS14	Soil	Paper mill	0.8 mm	0.8 mm	0.7 mm	-	1.1 mm
SL17	Wood	Wood warehouse	0.4 mm	-	-	1.8 mm	2.1 mm
SL18	Wood	Wood warehouse	1.9 mm	-	-	1.6 mm	0.7 mm
DY1	Rotten wood	Biodiversity Park	4.2 mm	3.6 mm	2.3 mm	1.9 mm	1.6 mm
DY3	Soil	Biodiversity Park	0.2 mm	0.8 mm	-	1.9 mm	1 mm

**Table 2:** GC-MS results of untreated and treated alkali lignin.

Compound	Control	10 Days	20 Days	30 Days	40 Days
13-(2Methoxyphenyl)tricyclo[8.2.2.24,7]hexadeca-1	+	−	−	−	−
Phenol, 2,4-bis(1,1-dimethylethyl)-, phosphite	+	+	+	−	−
Phenol,4,4’-butylidenebis[2-(1,1-dimethylethyl)-5-methyl-	+	−	−	−	−
7,9-Di-tert-butyl-1-oxaspiro (4,5) deca-6,9-diene-2,8-dione	+	+	+	+	+
n-hexadecanoic acid	−	+	+	+	+
oleic acid	−	−	−	+	+
Butylated hydroxytoluene	−	−	+	+	+
Dodecane, 2,6,11-trimethyl-	−	−	−	−	+
Octadecane	−	−	−	+	+
Dibutyl phthalate	−	−	+	+	+
Phenol, 3,5-dimethoxy-, acetate	−	−	+	−	−
Cyclohexanone, 2-(phenylmethylene)-	−	−	−	+	−

+ (Present), − (Absent).

**Table 3:** SEC results of untreated and treated Alkali lignin after 7 days of inoculation with *Aspergillus ochraceus* DY1.

Sample	RT	PMw	Mw	Mn	Mw/Mn	StartMw	EndMw	Total Area	Mz
**Treated**	7.803	7330.6	12,219.2	7310.5	1.67145	128,513.2	631.2	1.067712	27,989
**Control**	7.652	9653.1	15,776.2	7165.1	2.2018	152,729.7	1055.5	1.47431	35,651

**RT**: retention time, **PMw**: peak molecular weight, **Mw**: weight-average molecular weight, **Mn**: number-average molecular weight, **Mw/Mn**: polydispersity index (PDI), **StartMw**: starting molecular weight, **EndMw**: ending molecular weight, **Total Area**: the total area under the GPC curve, **Mz**: Z-average molecular weight.

## Data Availability

All available data are reported in the paper.
